# Effects of Metformin on CD133+ Colorectal Cancer Cells in Diabetic Patients

**DOI:** 10.1371/journal.pone.0081264

**Published:** 2013-11-21

**Authors:** Yanfei Zhang, Meiping Guan, Zongji Zheng, Qian Zhang, Fang Gao, Yaoming Xue

**Affiliations:** Department of Endocrinology & Metabolism, Nanfang Hospital, Southern Medical University, Guangzhou, Guangdong, China; University of Kentucky, United States of America

## Abstract

In diabetic patients complicated with colorectal cancer (CRC), metformin treatment was reported to have diverse correlation with CRC-specific mortality. In laboratory studies, metformin was reported to affect the survival of cancer stem cells (CSCs) in breast and pancreatic cancers and glioblastoma. Although cscs play a critical role in the resistance to 5-fluorouracil (5-FU) chemotherapy in CRC patients, the effect of metformin on cscs in CRC patients and the synergistic effect of metformin in combination with 5-FU on cscs are not reported. In the present study pathological examinations were performed in 86 CRC patients complicated with type 2 DM who had been divided into a metformin group and a non-metformin group. Comparisons regarding pathological type, incidence of metastasis, expression of CD133 and β-catenin were conducted between the two groups. We explored the synergistic effects of metformin in combination with 5-FU on the proliferation, cell cycle, apoptosis and the proportion of CD133+ cscs of SW620 human colorectal cancer cell lines. The results show that metformin treatment had reverse correlations with the proportion of patients with poorly differentiated adenocarcinoma, the proportion of CD133+ cscs in CRC patients with type 2 DM. Metformin enhanced the antiproliferative effects of 5-FU on CD133+ cscs in SW620 cells. These findings provide an important complement to previous study. Inhibition of the proliferation of CD133+ cscs may be a potential mechanism responsible for the association of metformin use with improved CRC outcomes in CRC patients with type 2 diabetes.

## Introduction

 Management of diabetic patients complicated with colorectal cancer (CRC) is a great challenge for clinicians. Epidemiologic studies have shown that diabetes mellitus (DM) is closely related to the incidence of cancers, especially gastrointestinal malignancy [1,2]. A meta-analysis of 15 studies involving a total of over 2.5 million people, showed that diabetes was associated with a 30% excess risk of CRC [3]. Moreover, diabetes is significantly associated with increased overall and CRC-specific mortality [4,5] while metformin (1,1-dimethyl biguanide hydrochloride), the most widely prescribed oral antidiabetic drug for type 2 DM [6,7], may decreased cancer risk and CRC-specific mortality in diabetic patients [8]. Accumulated evidence suggest that metformin might be a potential drug for the chemoprevention of CRC in diabetic patients. In our prior study, metformin inhibits the growth of SW-480 cells incubated with or without advanced glycation end products (AGEs) and down-regulates the expression of cyclin D1 and the telomerase activity [9,10]. The antineoplastic effects of metformin have been reported to be associated with activation of AMP-activated protein kinase (AMPK) signaling pathway, improvement of insulin resistance and hyperinsulinaemia [11,12].

 Most recently, another antineoplastic benefit of metformin was reported. It might inhibit the survival of cancer stem cells (CSCs, tumor-initiating stem-like cells: TISCs) in breast, and pancreatic cancers and glioblastoma in vitro [13,14]. As CSCs possess the potential to initiate and sustain tumor growth and metastasis, they are responsible for the resistance to chemotherapy and recurrence of cancers, in which Wnt/β-catenin signaling pathway may be involved [15,16]. CD133-positive (CD133+) cells separated from CRC exhibit the properties of CSCs, like self-renewal and high tumorigenic potential. In breast cancer, CD133 has been reported as a useful marker for predicting the effectiveness of chemotherapy and recurrence [17]. Similar roles of CD133 have also been identified in CRC. The high proportion of CD133+ cells was highly correlated with poor overall survival (OS) in CRC patients [18]. However, there is no research into the correlation between the metformin treatment and the proportion of CD133+ CSCs in CRC patients.

 What is more, there is no research either into the correlation between the metformin treatment and the 5-Fluorouracil (5-FU) chemotherapy. Metformin has recently been reported to have a synergistic effect in combination with some chemotherapy [19,20]. 5-FU, a first-line chemotherapeutic drug for CRC patients, is usually used in combination with other chemotherapeutic drugs to enhance the therapeutic efficacy, since resistance to 5-FU likely occurs in advanced CRC patients and often leads to the failure of chemotherapy [21]. Therefore, it needs to be explored whether metformin can be used in combination with 5-FU to enhance the antiproliferative effect of 5-FU on CRC. Considering the important role of CSCs in tumor progression we hypothesized that the positive role of metformin in CRC might be partially contributed to its antiproliferative effect on colorectal CSCs. 

 In order to clarify how metformin affects the pathogenesis and pathological progression of CRC with type 2 DM, we examined the associations of metformin with the pathological type and the incidence of metastasis of CRC in diabetic patients complicated with CRC and the antiproliferative effect of metformin on colorectal CSCs (CD133+) as well. In order to understand how metformin synergistically with 5-FU to affects the cellular behaviour of CRC, we examined the synergistic effects of metformin on the expression of CD133+ cells in combination with 5-FU in vitro.

## Materials and Methods

This study was approved by the ethics committee of Nanfang Hospital, Southern Medical University.

The ethics committee of Nanfang Hospital waived the need for patients' consent since the samples were procured from the tissue bank of department of pathology, Nanfang Hospital, Southern Medical University.

### 1.1.1 Patients and study design

 A total of 187 type 2 diabetic patients underwent resection of CRC at Nanfang Hospital, Southern Medical University in Guangzhou, China between January 2010 and June 2012. The medical records of these patients were retrieved for epidemiological investigation of their demographics and clinical characteristics. The use of other diabetic medications (sulfonylureas, thiazolidinediones, alpha-glucosidase inhibitors, insulin, and so on) was also investigated. The median age was 64 yrs old (ranging from 29 to 87 yrs old) and 119 (63.64%) of the patients were above 60 yrs old. 88 (47.06%) of the patients had newly diagnosed type 2 DM. The duration of DM less than 5 yrs and more than 5yrs accounted for 26.74% (50/187) and 22.99% (43/187) respectively. The others (6/187, 3.21%) had no clear information about the duration of DM. Finally, 86 patients with complete medical records were enrolled in this study and were divided into two groups according to the use of metformin. In the metformin group there were 36 patients who had used consistently metformin before CRC diagnosis and in the non-metformin group there were 50 patients. Pathologic specimens of 37 patients who had undergone neither chemotherapy nor radiotherapy before the resection of CRC were retrieved for immunohistochemical examination of the expressions of CD133 and β-catenin.

 The two groups were compared in terms of demographics, clinical characteristics and laboratory findings. Patient demographics and clinical characteristics included age at diagnosis, gender, duration of diabetes, body mass index (BMI), systolic/diastolic blood pressure (SBP/DBP). Laboratory findings included fasting plasma glucose (FPG), haemoglobin A1c (HbA1C), blood urine nitrogen (BUN), creatinine (CR), triglycerides (TG), total cholesterol (T-Chol), low-density lipoprotein cholesterol (LDL), very low-density lipoprotein cholesterol (VLDL) and high-density lipoprotein cholesterol (HDL), as well as the information pertaining to the CRC diagnosis, including histology, differentiation, and metastasis. The date of CRC diagnosis was defined as the day of definite pathologic diagnosis. 

### 1.1.2 Immunohistochemical Examinations

 Tissue samples from each patient of the 37 patients were fixed in formalin and embedded in paraffin. Next the paraffin sections were deparaffinized before, they were heated for 20 min at 105°C in antigen retrieval buffer (Jinqiao Zhongshan BioTech, Beijing, China). After blocking with 10% goat serum, the slides were incubated with primary monoclonal antibodies respectively against CD133 (BIOS, China, dilution 1:150) and β-catenin (Cell Signaling Technology, USA, dilution 1:200) overnight at 4°C followed by horseradish peroxidase-labeled secondary antibodies for 1 h at room temperature. And then, the slides were developed with diaminoben-zidine tetrahydrochloride (DAB) and counterstained with hematoxylin.

### 1.1.3 Immunohistochemical Assessment

 For each slide, at least 5 fields ( inside the tumor ) and >500 cells were analyzed with high power (×400 magnification) microscopy by two pathologists. Specimens were defined as positive for CD133 expression if tumor cells were distinctly stained by the anti-CD133 antibodies. The results were classified into two levels: < 10% and ≥10% CD133-positive cells.

 The expressions of β-catenin on the cell membrane and in the cytoplasm and nuclei were recorded. A >70% expression of β-catenin on the cell membrane was considered normal, recorded as negative (-), whereas a >10% expression of β-catenin in the cytoplasm and nuclei was considered abnormal, recorded as positive (+).

### 1.2.1 Cell lines and cell culture conditions

 The human colorectal cancer cell line SW620 was obtained from ATCC (USA) and maintained in RPMI-1640 medium supplemented with 10% fetal bovine serum (FBS; PPA, Austria) in a humidified atmosphere with 5% CO2 at 37°C. 5-FU and metformin were purchased from Sigma-Aldrich, St Louis, MO, USA. To assess the effects of metformin combined with 5-FU, the cells were treated with or without metformin (5mM) for 24h, and then washed with PBS, thereafter treated by 5-FU (5μΜ) for another 48h. 

### 1.2.2 Cell proliferation assays

 SW620 cells were seeded in 96-well plates before treatment with or without metformin followed by 5-FU for 24, 48, and 72 h respectively. Cell proliferation was measured with the 3-(4,5-dimethylthiazol-2-yl)-2,5-diphenyltetrazolium bromide (MTT; 5 mg/ml; Sigma, MO, USA) assay. After appropriate treatment time, MTT was added to a final concentration of 1mg/mL, and the reaction mixture was incubated for 3 hours at 37°C. The resulting crystals were dissolved in 0.04% HCl in isopropanol and the absorbance was read at 562 nm. Each treatment was conducted in triplicate and each experiment was repeated twice. 

### 1.2.3 Cell cycle assays

 SW620 cells were pre-treated with metformin for 24 h or not, and then treated with 5-FU for 48 h. Cells were harvested at an exponential growth phase, and single-cell suspensions containing 1x106 cells were fixed with 70% alcohol. Cell cycle was monitored using propidium iodide (PI) staining of nuclei. The fluorescence of DNA-bound PI in cells was measured with a flow-cytometry (BD Biosciences). The results were analyzed with ModFit 3.0 software (Verity Software House, Topsham, ME). The experiment was performed three times. The ratio of cells in the G0/G1, intra-S, and G2/M phases were expressed as mean ± SD.

### 1.2.4 Assessment of apoptosis

 Apoptosis was detected by flow cytometry via examination of the altered plasma membrane phospholipid packing by lipophilic dye Annexin V. Briefly, treated cells were harvested and washed twice with PBS before resuspended at a concentration of 1×106 cells/mL in binding buffer according to the manufacturer’s instructions (AnnexinV: FITC Apoptosis Detection Kit, BD Pharmingen, CA, USA). Thereafter, 5 μL of Annexin V-FITC and 5 μL of propidium iodide (PI) were added into 100 μL of cell suspension and incubated for 15 min at room temperature in the dark. After adding 400 μL of binding buffer, labeled cells were counted within 30 min by FACS Calibur flow cytometer (Becton-Dickinson, Mountain View, Calif). Early apoptosis cells (Annexin V-positive, PI-negative), necrotic/late apoptosis cells (double positive), as well as living cells (double negative) were measured before subsequent analysis by Cell Quest software (Becton-Dickinson). 

### 1.2.5 Detection of CD133 positive cells

 After SW620 cells were prepared and treated as described, the tumor cells were collected and stained with anti-CD133 antibody (mouse monoclonal IgG, 1: 10, Milteny Biotec) or IgG1 isotype control antibody (Milteny Biotec) for 30 min in the dark at 2-8°C. CD133 staining was analyzed by flow cytometry (Becton Dickinson, San Jose, CA). The experiments were performed in triplicate. The ratio of CD133+ cells was expressed as mean ± SD.

### 1.3 Statistical analysis

 Data were presented as the mean + Standard deviation (SD) and analyzed using SPSS v.16.0 statistical software (Abbott Laboratories, North Chicago, IL). One-way analysis of variance (ANOVA) and then unpaired Student’s *t*-test were used to determine the effects of different variables. The associations between the expressions of CD133 and β-catenin and clinicopathological parameters were analyzed with the use of Pearson’s chi-square (χ^2^) test and Fisher’s exact test. *P* value less than 0.05 was considered to be statistically significant.

## Results

### 2.1.1 Patient demographics and clinical characteristics

 Patient demographics and clinical characteristics are summarized in Table 1. There were no significant differences between the metformin and non-metformin groups regarding age at diagnosis, blood pressure, BMI, duration of diabetes, FPG, HbA1c, BUN, CR, ALB, TG, T-Chol, HDL, or VLDL levels at the baseline, but the LDL level was significantly lower in the metformin group than in the non-metformin group (p=0.024). As for the pathologic characteristics between the two groups, the proportion of patients with poorly differentiated adenocarcinoma in the metformin group was significantly lower than in the non-metformin group (2.78% vs 16.0%, p=0.048). Furthermore, the distant metastasis rate in the metformin group was significantly lower than in the non-metformin group (5.60% vs 21.6%, p=0.035). However, no significant difference was observed between the two groups in the lymph node metastasis (table 2).

**Table 1:** Patient demographics and clinical characteristics at baseline.

	Metformin group (n=36)	Non-metformin group (n=50)	p
Age at diagnosis (yrs)	66.19±9.88	63.22±9.94	0.725
DM duration (yrs)	4.99±3.68	5.75±4.70	0.334
BMI (kg/m^2^)	22.39±3.41	25.09±3.45	0.894
SBP (mmHg)	144.72±21.82	137.66±19.23	0.199
DBP (mmHg)	80.11±9.72	77.98±17.72	0.306
BUN (mmol/L)	6.92±9.83	6.42±3.78	0.430
CR (mmol/L)	92.61±69.21	97.94±108.49	0.253
ALB (g/L)	34.96±6.93	33.71±5.76	0.063
FBG (mmol/L)	8.63±2.09	9.27±2.95	0.080
HbA1c (%)	6.78±1.06	7.61±1.99	0.083
TG (mmol/L)	1.75±1.28	2.51±3.07	0.132
T-Chol (mmol/L)	4.44±0.83	5.42±4.26	0.173
HDL (mmol/L)	1.25±0.25	1.27±0.85	0.203
LDL (mmol/L)	2.27±0.52	2.41±1.07	0.024
VLDL (mmol/L)	0.88±0.49	0.95±0.54	0.741

**Table 2:** Pathologic characteristics and metastasis in two groups.

		Metformin group (n=36)	Non-metformin group (n=50)	p
Pathologic characteristics	Well differentiated adenocarcinoma	12(33.33%)	13(26.0%)	0.460
	moderately differentiated adenocarcinoma	21(58.33%)	22(44.0%)	0.190
	poorly differentiated adenocarcinoma	1 (2.78%)	8(16.0%)	0.048
	mucinous adenocarcinoma	2（5.56%,)	6(12.0%,)	0.310
	signet ring cell carcinoma	0	1(2.0%,)	
Metastasis	lymph node	7(19.44%)	14(28.0%)	0.257
	distant	2(5.60%)	11(21.6%)	0.035

### 2.1.2 Expressions of CD133 and β-catenin in CRC specimens

**Figure 1:**
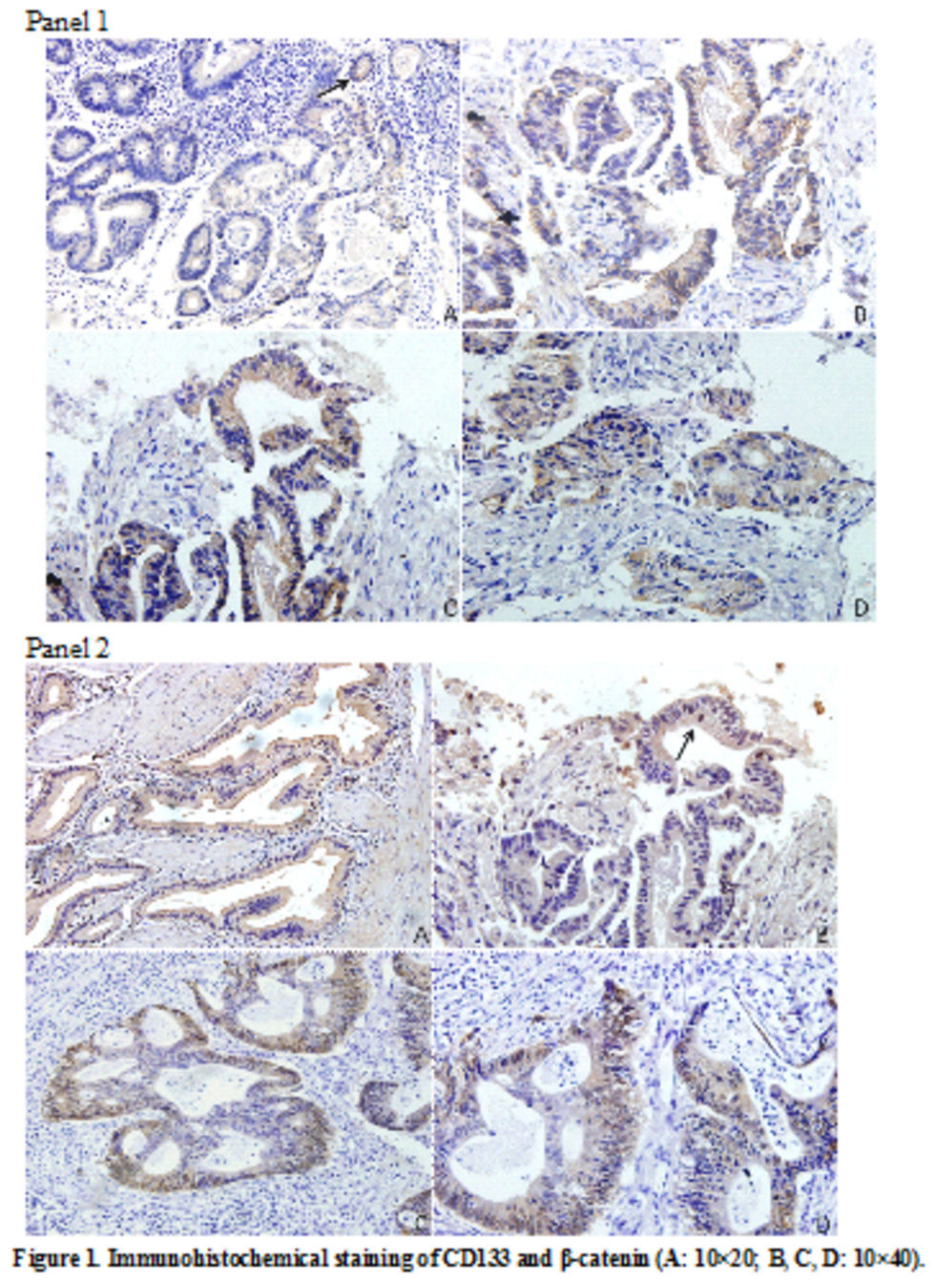
Panel 1: the CD133 antibodies stained intensely at the membrane and in the cytoplasm of cancer cells. (A): weakly positive or focally positive staining in 10% of cells; (B): moderately or strongly positive-staining involving 10% or more of the cells; (C) and (D): the staining of CD133 on the luminal surface and the basal surface of cancer cells. Panel 2: (A): cytoplasmic or nuclear expression of β-catenin was absent; (B): nuclear expression of β-catenin was less than 10% of the cancer cells; (C) and (D): widespread nuclear accumulation of β-catenin.

 Immunohistochemical patterns of CD133 and β-catenin expressions were analyzed in CRC specimens. CD133 brownish signals were observed on the cell membrane, especially on its luminal and basal surface. In general, higher staining intensity of CD133 indicates higher percentage of CD133+ tumor cells (Figure 1, panel 1). In the metformin group, 15 of the 19 CRC specimens (78.9%) contained less than 10% CD133-positive tumor cells while the other 4 (21.1%) contained more than 10% CD133-positive tumor cells. Although half of the CRC specimens (9/18, 50.0%) in the non-metformin group contained more than 10% CD133-positive tumor cells, *the difference between the two groups did not reach a significant level* (*p=0.065*). (table 3)

**Table 3:** The expressions of CD133 and β-catenin in two groups.

Positive expression*	Metformin group(n=19)	Non-metformin group(n=18)	p-Value
CD133	4 (21.1%)	9 (50.0%)	0.065
β-catenin	7 (36.8%)	13 (72.2%)	0.031

* greater than or equal to 10% expression of CD133 in the cells was recorded as positive; >10% expression of β-catenin in the cytoplasm and nuclei was recorded as positive.

Expression of β-catenin was clearly evident at the cell-cell boundaries and cytoplasm in a majority of CRC specimens, while in some of the cells nuclear accumulation of β-catenin protein was observed (Figure 1, panel 2). There was a significant difference between the two groups in the positive rate of β-catenin protein expression (p=0.031). The nuclear accumulation rate of β-catenin in more than 10 % of the tumor cells was significantly lower in the metformin group than in the non-metformin group (7/19, 36.8% vs 13/18, 72.2%; p=0.031). (table 3)

There is a significantly positive correlation between CD133 and β-catenin expression (p=0.028). *The expression of CD133 is correlated with the pathological type of CRC but they did not reach a significant level (p=0.056*). There is no significant correlation between β-catenin and the pathological type of CRC (p=0.335).

### 2.2.1 Antiproliferative effect of 5-FU alone and in combination with metformin on SW620 cells

**Figure 2:**
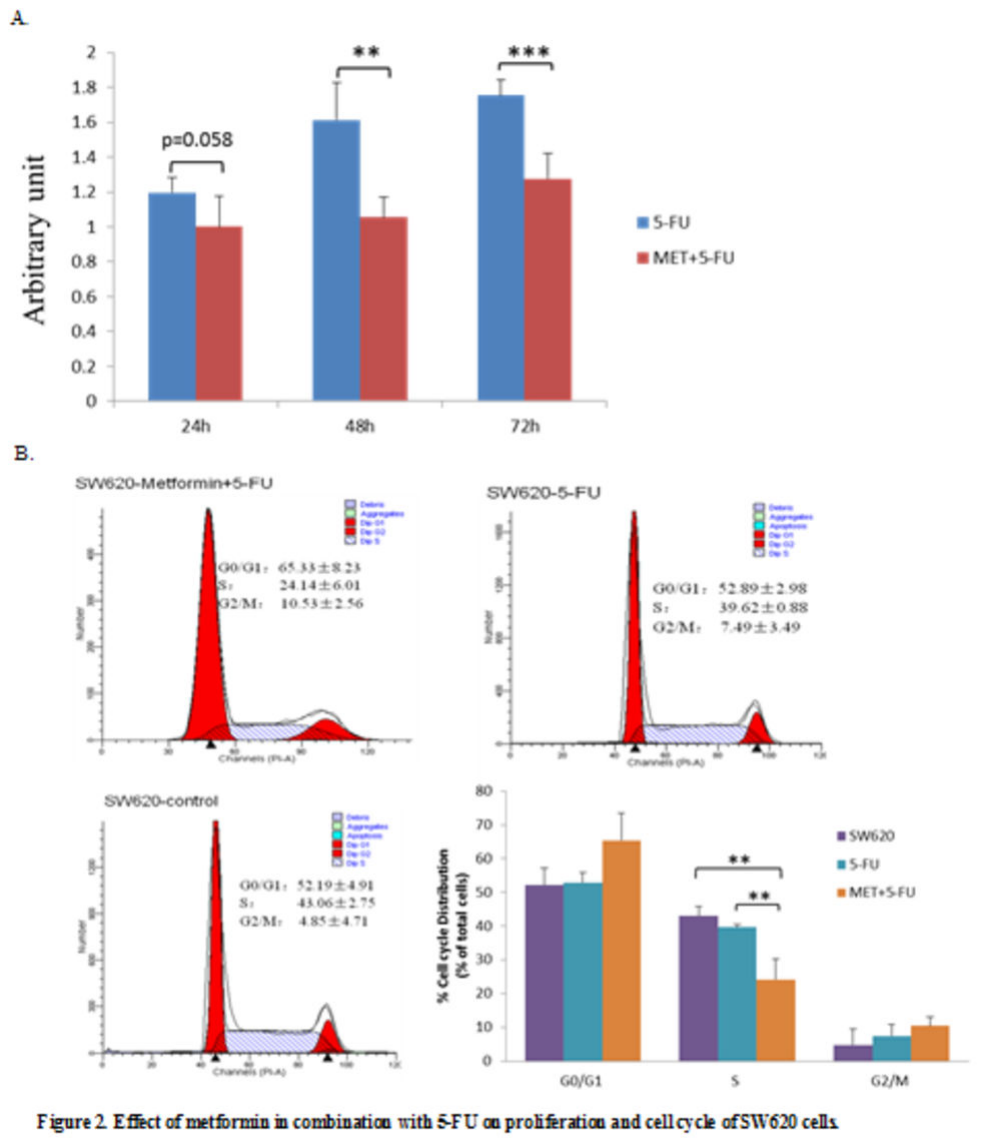
A: The proliferation was insignificantly lower at the 24 h but significantly lower at the 48 h and 72 h in the MET+5-FU group than in the 5-FU group respectively (1.019±0.181 / 24 h vs 1.218 ± 0.090 / 24 h, p=0.058; 1.075 ± 0.118 / 48 h vs 1.644 ± 0.219 / 48 h, p=0.001; 1.299 ± 0.147 / 72 h vs 1.786±0.109 / 72 h, p<0.001). B: The proportion of SW620 cells in either G0/G1 or G2/M phase was not obviously changed among the three groups (p=0.06 and p=0.248 respectively), but the metformin pretreatment significantly reduced the proportion of SW620 cells in S phase (24.14 ± 6.01 vs 39.62 ± 0.88, P=0.003). **, p<0.01 for comparisons of MET+5-FU treatment respectively with 5-FU treatment and control.

 In vitro, SW620 cells were treated respectively with 5-FU alone and with a combination of 5-FU and metformin. The proliferation of SW620 cells in both groups increased with the time extending. We observed that metformin pretreatment followed by 5-FU treatment significantly inhibited the proliferation of the SW620 cells as compared with the treatment of 5-FU alone (1.019±0.181 vs 1.218 ± 0.090, p=0.058 for 24 h exposure; 1.075 ± 0.118 vs 1.644 ± 0.219, p=0.001 for 48 h exposure; 1.299 ± 0.147 vs 1.786±0.109, p<0.001 for 72 h exposure). (Figure 2A)

### 2.2.2 Synergistic effect of metformin and 5-FU on the cell cycle of SW620 cells

 To investigate the effect of metformin combined with 5-FU on the cell cycle, we pretreated SW620 cells with metformin for 24 h prior to 5-FU treatment. Thereafter, the proportions of SW620 cells in G0/G1, S, and G2/M phases were analyzed by flow cytometry. We observed that there were no significant difference among the three groups in the proportion of SW620 cells in G0/G1 or G2/M phase (p>0.05), while the metformin pretreatment significantly reduced that in S phase as compared with the 5-FU alone treatment (24.14 ± 6.01% vs 39.62 ± 0.88%, p=0.003) (Figure 2B).

### 2.2.3 Effect of MET+5-FU treatment on apoptosis of SW620 cells

**Figure 3:**
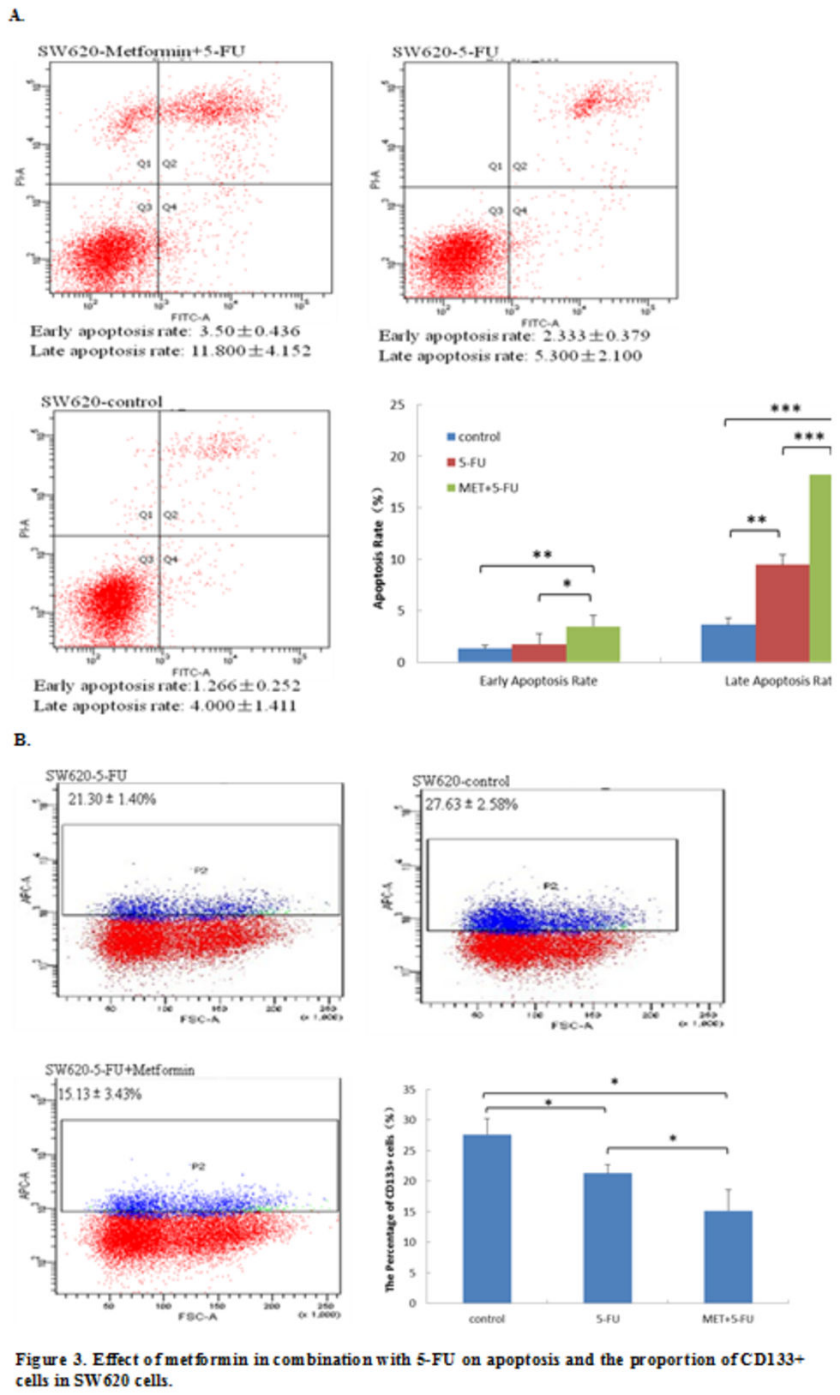
A: MET+5-FU treatment significantly increased the percentage of annexin V+ apoptotic cells as compared with 5-FU alone treatment (Early apoptosis rate: 3.50 ± 0.44 % vs 2.33 ± 0.38%, p=0.029; Late apoptosis rate: 11.80 ± 4.15 % vs 5.30 ± 2.10 %, p=0.000). *, p<0.05 and ***, p<0.001 for comparisons between the MET+5-FU group and the 5-FU alone group. B: The proportion of CD133 + cells was significantly decreased by the MET+5-FU treatment as compared with 5-FU alone treatment (15.13± 3.43 vs 21.30 ± 1.40; p=0.027). Values were given as mean ± SD.

 The percentage of apoptotic cells was significantly increased by metformin pretreatment followed by 5-FU treatment as compared with 5-FU alone treatment (Early apoptosis rate: 3.50 ± 0.44% vs 2.33 ± 0.38%, p=0.029; Late apoptosis rate: 11.80 ± 4.15% vs 5.30 ± 2.10 %, p<0.001) (Figure 3A). 

### 2.2.4 Effects of MET+5-FU treatment on proportion of CD133+ cells

 The proportion of CD133+ cells was determined by flow cytometry. 27.63 ± 2.58% of the SW620 cells expressed the membrane antigen CD133 and both the 5-FU alone and MET+5-FU treatments significantly decreased the proportion of CD133-positive cells. Furthermore, metformin pretreatment for 24 h followed by 5-FU treatment for 48 h significantly reinforced the inhibiting effect of 5-FU alone treatment on the proportion of CD133-positive cells (15.13 ± 3.43 vs 21.30 ± 1.40; p=0.027) (Figure 3B). 

## Discussion

In the CRC patients complicated with type 2 diabetes, the present study showed that the distant metastasis rate in the metformin group was significantly lower than in the non-metformin group (5.60% vs 21.6%, p=0.035) and the proportion of patients with poorly differentiated adenocarcinoma was significantly smaller in the metformin group than in the non-metformin group (2.78% vs 16.0%, p=0.048). These results show another benefit of metformin in CRC patients. In the past decade, epidemiologic studies and large, randomized controlled trials (RCT), such as ADOPT (A Diabetes Outcome Progression Trial) and RECORD (Rosiglitazone Evaluated for Cardiovascular Outcomes and Regulation of Glycaemia in Diabetes), have suggested a reverse correlation between metformin use and cancer risk, especially the risk of colorectal cancers (CRC), as compared with other antidiabetic treatments [22,23]. Metformin treatment resulted in a reduced risk of overall mortality and CRC-specific mortality in CRC patients with diabetes [24]. A prospectively randomized study showed that metformin treatment reduced the number of rectal aberrant crypt foci (ACF), an endoscopic surrogate marker of CRC, in patients without diabetes [25]. In several cancer cell lines and animal models, metformin treatment exerts antiinvasive and antimetastasis effects [26-29]. However, few studies addressed the correlations of metformin treatment with cancer metastasis and with the pathologic type of cancers in human populations. The present study is a tentative attempt of ours to explore these questions. The strength of this research is our pathological observation of a subgroup of CRC patients with concomitant type 2 diabetes who were receiving metformin treatment. We found solid pathological evidence supporting a remarkable association of metformin with improved outcomes in such patients, which triggered our further study into some of the mechanisms underlying the association. 

 In our prior study, the proliferation of SW480 cells were significantly inhibited by metformin treatment in a dose- and time-dependent manner which may due to the down-regulation of cyclin D1 expression and the telomerase activity by metformin [9,10]. In the present study, we show that metformin have synergistic effects with 5-FU on the proliferation and cell cycle of SW620 cells. The significant growth inhibitory and pro-apoptotic effects of metformin on cancer cells probably due to the activation of LKB1/AMPK and the improvement of insulin resistance [19,20,30]. In addition, Ras/Raf/MAPK pathway and reductive glutamine metabolism have also been reported to play a role in the antineoplastic effect of metformin [19]. Most recently, metformin was reported to affect the survival of CSCs in several cancer cell lines [13,14]. CSCs have been identified as therapeutic targets for tumor progression [31], because they play an important role in the genesis and recurrence of tumors, metastasis and the resistance to chemotherapy as a result of two critical properties: self-renewal capacity and differentiate potential into unlimited heterogeneous populations of cancer cells. Subsequently, we explored the effect of metformin treatment on the colorectal CSCs in CRC patients, using their pathological specimens. We thus found a reverse correlation of metformin treatment with CD133+ CSCs in CRC patients. CD133 has been believed to be the most robust surface marker for colorectal CSCs. The patients with CD133-high expression seemed to have a much lower rate of 5-year OS and more chances of T3, 4 tumor invasion, positive N and vascular invasion than those with CD133-low expression [32]. 

 After our finding of the association of metformin treatment with the proportion of CD133+ CSCs in CRC of these patients, we further investigated the synergistic effect of metformin plus 5-FU treatment on the proportion of CD133+ cells and the expression of β-catenin protein in SW620 cells. We revealed that metformin in combination with 5-FU significantly decreased both the proportion of CD133+ cells and the expression of β-catenin protein, indicating metformin might have a synergistic antineoplastic effect on CRC by inhibting the proliferation of CSCs via the β-catenin pathway. A similar antineoplastic effect of metformin has been reported in breast cancer [20]. Although 5-FU is the most common chemotherapeutic agent in the treatment of CRC, the resistance to this drug is very common in chemotherapy of CRC patients. The activation of Wnt/β-catenin pathway in CD133 (+) colorectal CSCs may be responsible for the resistance of the chemotherapy in CRCs [15,16,33]. Recently, AMPK activators were reported to suppress cervical cancer cell growth by inhibiting Wnt/β-catenin pathway [34]. Inhibition of Wnt/β-catenin pathway by AMPK may be responsible for the synergistic effect of metformin in combination with 5-FU on the proliferation of colorectal CSCs. It is noticeable that we found the combined treatment of metformin plus 5-FU had a significantly better antineoplastic effect than the treatment of 5-FU alone, suggesting that the combined treatment might be a potential standard chemotherapy for CRC patients, particularly for those who are highly resistant to 5-FU. Of course, further researches, both clinical trials and laboratory experiments, are needed to verify this assumption. 

 In conclusion, our study further confirms that metformin is not only an antidiabetic drug but also a potential anticancer medication. Our results reveal reverse correlations of metformin treatment with the incidence of distant metastasis and poorly differentiated adenocarcinoma, the positive rate of CD133 and the expression of β-catenin protein in CRC patients with type 2 DM. We also demonstrate the synergistic effects of metformin in combination with 5-FU on the cell biology and proportion of CD133+ cells in vitro. However, the mechanism of metformin interfering with the proliferation of CSCs remains underdetermined. In the future, prospective RCTs are required to investigate the effect of metformin in combination with 5-FU on the prognosis of CRC patients and the underlying mechanisms should be further explored. 

## References

[1] Lee MS, Hsu CC, Wahlqvist ML, Tsai HN, Chang YH et al. (2011) Type 2 diabetes increases and metformin reduces total, colorectal, liver and pancreatic cancer incidences in Taiwanese: a representative population prospective cohort study of 800,000 individuals. BMC Cancer, 11: 20-. doi:10.1186/1471-2407-11-20. PubMed: 21241523. 21241523 PMC3031263

[2] Chiu CC, Huang CC, Chen YC, Chen TJ, Liang Y et al. (2013) Increased Risk of Gastrointestinal Malignancy in Patients with Diabetes Mellitus and Correlations with Anti-Diabetes Drugs: A Nationwide Population-based Study in Taiwan. Intern Med 52: 939-946. doi:10.2169/internalmedicine.52.8276. PubMed: 23648711. 23648711

[3] Larsson SC, Orsini N, Wolk A (2005) Diabetes mellitus and risk of colorectal cancer: a meta-analysis. J Natl Cancer Inst 97: 1679-1687. doi:10.1093/jnci/dji375. PubMed: 16288121. 16288121

[4] Bella F, Minicozzi P, Giacomin A, Crocetti E, Federico M et al. (2013) Impact of diabetes on overall and cancer-specific mortality in colorectal cancer patients. J Cancer Res Clin Oncol [Epub ahead of print]. PubMed: 23633003. 10.1007/s00432-013-1439-8 PMC11824221 23633003

[5] Coughlin SS, Calle EE, Teras LR, Petrelli J, Thun MJ (2004) Diabetes mellitus as a predictor of cancer mortality in a large cohort of US adults. Am J Epidemiol 159: 1160–1167. doi:10.1093/aje/kwh161. PubMed: 15191933. 15191933

[6] Nathan DM, Buse JB, Davidson MB, Ferrannini E, Holman RR et al. (2009) American Diabetes Association; European Association for Study of Diabetes. American Diabetes Association; European Association for Study of Diabetes. Medical management of hyperglycemia in type 2 diabetes: a consensus algorithm for the initiation and adjustment of therapy: a consensus statement of the American Diabetes Association and the European Association for the Study of Diabetes. Diabetes Care 32: 193-203. doi:10.2337/dc08-9025. PubMed: 18945920. 18945920 PMC2606813

[7] Nathan DM, Buse JB, Davidson MB, Ferrannini E, Holman RR et al. (2009) American Diabetes Association; European Association for the Study of Diabetes. American Diabetes Association; European Association for the Study of Diabetes. Medical management of hyperglycaemia in type 2 diabetes mellitus: a consensus algorithm for the initiation and adjustment of therapy: a consensus statement from the American Diabetes Association and the European Association for the Study of Diabetes. Diabetologia 52: 17–30. doi:10.1007/s00125-008-1157-y. PubMed: 18941734. 18941734

[8] Noto H, Goto A, Tsujimoto T, Noda M (2012) Cancer Risk in Diabetic Patients Treated with Metformin: A Systematic Review and Meta-analysis. PLOS ONE 7(3): e33411. doi:10.1371/journal.pone.0033411. PubMed: 22448244. 22448244 PMC3308971

[9] Zhou XZ, Xue YM, Zhu B, Sha JP (2010) Effects of metformin on proliferation of human colon carcinoma cell line SW-480. NAN Fang Yi Ke da Xue Xue Bao ( J South Med Univ); 30(8): 1935-1942. 20813708

[10] Zhou XZ, Xue YM, Fang Gao, Bo Zhu, Guan MP (2012) Effect and mechanism of metformin on AGEs-induced proliferation of colon carcinoma cell line SW-480. Chin J Diabetes; 20(7): 546-548.

[11] Zakikhani M, Dowling R, Fantus IG, Sonenberg N, Pollak M (2006) Metformin is an AMP kinase-dependent growth inhibitor for breast cancer cells. Cancer Res 66: 10269–10273. doi:10.1158/0008-5472.CAN-06-1500. PubMed: 17062558. 17062558

[12] Rizos CV, Elisaf MS (2013) Metformin and cancer. Eur J Pharmacol 705: 96–108. doi:10.1016/j.ejphar.2013.02.038. PubMed: 23499688. 23499688

[13] Gou S, Cui P, Li X, Shi P, Liu T et al. (2013) Low Concentrations of Metformin Selectively Inhibit CD133(+) Cell Proliferation in Pancreatic Cancer and Have Anticancer. Action - PLOS ONE 8(5): e63969]. doi:10.1371/journal.pone.0063969. 23667692 PMC3648476

[14] Würth R, Pattarozzi A, Gatti M, Bajetto A, Corsaro A et al. (2013) Metformin selectively affects human glioblastoma tumor-initiating cell viability: A role for metformin-induced inhibition of Akt. Cell Cycle 12(1): 145-156. doi:10.4161/cc.23050. PubMed: 23255107. 23255107 PMC3570504

[15] Deng YH, Pu XX, Huang MJ, Xiao J, Zhou JM et al. (2010) 5-Fluorouracil upregulates the activity of Wnt signaling pathway in CD133-positive colon cancer stem-like cells. Chin J Cancer 29(9): 810-815. doi:10.5732/cjc.010.10134. PubMed: 20800023. 20800023

[16] Kwan HT, Chan DW, Cai PC, Mak CS, Yung MM et al. (2013) AMPK activators suppress cervical cancer cell growth through inhibition of DVL3 mediated Wnt/β-catenin signaling activity. PLOS ONE 8(1): e53597. doi:10.1371/journal.pone.0053597. PubMed: 23301094. 23301094 PMC3534705

[17] Aomatsu N, Yashiro M, Kashiwagi S, Takashima T, Ishikawa T et al. (2012) CD133 is a useful surrogate marker for predicting chemosensitivity to neoadjuvant chemotherapy in breast cancer. PLOS ONE 7(9): e45865. doi:10.1371/journal.pone.0045865. PubMed: 23049880. 23049880 PMC3457956

[18] Chen S, Song X, Chen Z, Li X, Li M et al. (2013) CD133 Expression and the Prognosis of Colorectal Cancer: A Systematic Review and Meta-Analysis. PLOS ONE 8(2): e56380. doi:10.1371/journal.pone.0056380. PubMed: 23409180. 23409180 PMC3569427

[19] Morgillo F, Sasso FC, Della Corte CM, Vitagliano D, D'aiuto E et al. (2013) Synergistic effects of metformin treatment in combination with gefitinib, a selective EGFR tyrosine kinase inhibitor, in LKB1 wild-type NSCLC cell lines. Clin Cancer Res [Epub ahead of print]. PubMed: 23695170. 10.1158/1078-0432.CCR-12-2777 23695170

[20] Cufi S, Corominas-Faja B, Vazquez-Martin A, Oliveras-Ferraros C, Dorca J et al. (2012) Metformin-induced preferential killing of breast cancer initiating CD44+CD24-/low cells is sufficient to overcome primary resistance to trastuzumab in HER2+ human breast cancer xenografts. Oncotarget 3(4): 395-398. PubMed: 22565037. 22565037 10.18632/oncotarget.488 PMC3380574

[21] Kim SL, Kim SH, Trang KT, Kim IH, Lee SO et al. (2013) Synergistic antitumor effect of 5-fluorouracil in combination with parthenolide in human colorectal cancer. Cancer Lett 335(2): 479-486. doi:10.1016/j.canlet.2013.03.007. PubMed: 23507557. 23507557

[22] Chung HH, Moon JS, Yoon JS, Lee HW, Won KC (2013) The Relationship between Metformin and Cancer in Patients with Type 2. Diabetes - Diabetes Metab: 125 131. 10.4093/dmj.2013.37.2.125 PMC3638223 23641353

[23] Zhang ZJ, Zheng ZJ, Kan H, Song Y, Cui W et al. (2011) Reduced risk of colorectal cancer with metformin therapy in patients with type 2 diabetes: a meta-analysis. Diabetes Care 34(10): 2323-2328. doi:10.2337/dc11-0512. PubMed: 21949223. 21949223 PMC3177711

[24] Lee JH, Kim TI, Jeon SM, Hong SP, Cheon JH (2012) The effects of metformin on the survival of colorectal cancer patients with diabetes mellitus. Int J Cancer 131: 752–759. doi:10.1002/ijc.26421. PubMed: 21913184. 21913184

[25] Hosono K, Endo H, Takahashi H, Sugiyama M, Sakai E et al. (2010) Metformin suppresses colorectal aberrant crypt foci in a short-term clinical trial. Cancer. Prev Res (Phila) 3(9): 1077-1083. doi:10.1158/1940-6207.CAPR-10-0186. 20810669

[26] Rattan R, Graham RP, Maguire JL, Giri S, Shridhar V (2011) Metformin suppresses ovarian cancer growth and metastasis with enhancement of cisplatin cytotoxicity in vivo. Neoplasia 13(5): 483-491. PubMed: 21532889. 21532889 10.1593/neo.11148 PMC3084625

[27] Tan BK, Adya R, Chen J, Lehnert H, Cassia Sant LJ, et Tan BK, Adya R, Chen J, Lehnert H, Cassia Sant. LJ, Randeva HSal (2011) Metformin treatment exerts antiinvasive and antimetastatic effects in human endometrial carcinoma cells. J Clin Endocrinol Metab 96(3): 808-816 doi:10.1210/jc.2010-1803. PubMed: 21190977. doi:10.1210/jc.2010-1803. PubMed: 21190977.

[28] Vazquez-Martin A, Oliveras-Ferraros C, Cufí S, Del Barco S, Martin-Castillo B et al. (2011) The anti-diabetic drug metformin suppresses the metastasis-associated protein CD24 in MDA-MB-468 triple-negative breast cancer cells. Oncol Rep 25(1): 135-140. PubMed: 21109968. 21109968

[29] Hwang YP, Jeong HG (2010) Metformin blocks migration and invasion of tumour cells by inhibition of matrix metalloproteinase-9 activation through a calcium and protein kinase C alpha-dependent pathway: phorbol-12-myristate-13-acetate-induced/extracellular signal-regulated kinase/activator protein-1. Br J Pharmacol 160(5): 1195-1211. doi:10.1111/j.1476-5381.2010.00762.x. PubMed: 20590612. 20590612 PMC2936028

[30] Pierotti MA, Berrino F, Gariboldi M, Melani C, Mogavero A et al. (2013) Targeting metabolism for cancer treatment and prevention: metformin, an old drug with multi-faceted effects. Oncogene 32: 1475-1487. doi:10.1038/onc.2012.181. PubMed: 22665053. 22665053

[31] Bao B, Ahmad A, Azmi AS, Ali S, Sarkar FH. (2013) Overview of Cancer Stem Cells (CSCs) and Mechanisms of Their Regulation: Implications for Cancer Therapy. Curr. Protoc. Pharmacol Chapter 14 Unit14 25 10.1002/0471141755.ph1425s61 PMC3733496 23744710

[32] Chen Shicai, Song Xinming, Chen Zhihui, Xinxin Li Mingzhe; Chen et al. (2013) CD133 Expression and the Prognosis of Colorectal Cancer: A Systematic Review and Meta-Analysis. PLOS ONE 8(2): e56380. doi:10.1371/journal.pone.0056380. PubMed: 23409180. Available online at: 10.1371/journal.pone.0056380 Available online at: PubMed: 23409180 23409180 PMC3569427

[33] Zhu L, Gibson P, Currle DS, Tong Y, Richardson RJ et al. (2009) Prominin 1 marks intestinal stem cells that are susceptible to neoplastic transformation [J]. Nature 457(7229 ): 603-607. doi:10.1038/nature07589. PubMed: 19092805. 19092805 PMC2633030

[34] Horst D, Kriegl L, Engel J, Jung A, Kirchner T (2009) CD133 and nuclear beta-catenin: the marker combination to detect high risk cases of low stage colorectal cancer. Eur J Cancer 45(11): 2034-2040. doi:10.1016/j.ejca.2009.04.004. PubMed: 19403300. 19403300

